# Implementing specialised vestibular physiotherapy in an emergency department: a process evaluation

**DOI:** 10.1186/s43058-022-00313-2

**Published:** 2022-06-11

**Authors:** Kelvin Ip, Melanie Lloyd, Allison Luscombe, Danielle Hitch

**Affiliations:** 1 Allied Health, Western Health, Sunshine Hospital, 176 Furlong Road, St. Albans, VIC 3021 Australia; 2 Centre for Medicine Use and Safety, Monash University, 407 Royal Parade, Parkville, 3052 VIC Australia; 3 Occupational Science and Therapy, Deakin University, Waterfront Campus, 1 Gheringhap Street, Geelong, VIC 3217 Australia

**Keywords:** Physiotherapy, Vestibular rehabilitation, Benign peripheral positional vertigo, Dizziness, Emergency department, Allied health, Implementation

## Abstract

**Background:**

Dizziness and vertigo-like symptoms, often caused by common peripheral vestibular disorders such as benign paroxysmal positional vertigo (BPPV), may significantly impact function and quality of life. These symptoms often result in emergency department (ED) presentations. Evidence-based clinical practice guidelines strongly recommend using physical assessment and treatment manoeuvres for the assessment, diagnosis and treatment of these symptoms. This study aimed to evaluate the process of implementing specialised vestibular physiotherapy (SPV) in an emergency department from the clinician’s perspective.

**Methods:**

This implementation study utilised a retrospective mixed-methods process evaluation to understand how SVP operated in an Australian emergency department. The i-PARiHS framework was embedded within the methodology and analytical approach of the study to ensure a comprehensive approach closely aligned to implementation science. Nine clinicians retrospectively completed the Organisational Readiness for Change Assessment (ORCA), Acceptability of Intervention Measure (AIM), Intervention Appropriateness Measure (IAM) and Feasibility of Intervention Measure (FIM). Seven clinicians also participated in a focus group or interview.

**Results:**

A range of barriers and facilitators to the implementation process were identified by participants, some of which spanned multiple domains of the i-PARiHS framework. Relationships with service leaders, champions and medical staff were pivotal facilitators to implementation, along with a generally held perception that SVP was acceptable and feasible. The main barrier identified was a lack of capacity to deliver and facilitate this innovation within the physiotherapy workforce and the broader multidisciplinary recipients.

**Conclusions:**

This study demonstrates that the process of implementing an SVP service in an ED context was generally well-received by clinicians but also involved some challenges and barriers. Services looking to implement SVP in the ED should aim to build stakeholder relationships; develop a shared vision with clear goals and intended outcomes; embed the innovation in organisation processes, procedures and policies; and increase workforce capacity to deliver and facilitate SVP to guide their approach to this innovation.

**Supplementary Information:**

The online version contains supplementary material available at 10.1186/s43058-022-00313-2.

## Contribution to the literature

- Emerging evidence supports the use of specialised physiotherapy for peripheral vestibular disorders. However, implementation determinants, context and processes (particularly in emergency department contexts) were unknown.
- In reference to the Integrated Promoting Action on Research Implementation in Health Services (i-PARiHS) framework, the key implementation characteristics for clinicians are strong interpersonal and communication skills and the clinical knowledge and practical skills to provide specialised vestibular physiotherapy (SVP).
- Barriers posed by varying terminology for symptoms and innovations between patients and multidisciplinary clinicians emerged as an important priority for action.
- This study describes the experience of implementing SVP from the perspective of multiple clinicians, which may guide the broader dissemination of this innovation.

## Background

A common cause of dizziness and vertigo presentation to emergency departments (ED) is benign paroxysmal peripheral vertigo (BPPV), which is defined as disruption to the vestibular system caused by calcium crystals within the inner ear being dislodged into the semi-circular canals [1]. Clinical practice guidelines facilitate and recommend routine physical manoeuvres in the clinical context to assess and diagnose BPPV [2, 3]. Failure to accurately diagnose and treat BPPV during an initial presentation can result in adverse outcomes for patients, including inappropriate use of vestibular suppressants and other medications, increased risk of falls, ongoing disruption of daily activities and decreased quality of life [4–6].

Specialised vestibular physiotherapy (SVP) utilises manoeuvres like the Dix-Hallpike test (DHT) and supine roll test (SRT) and interventions such as canalith repositioning ﻿techniques (CRT) to assess and treat BPPV [7]. Treatment with a CRT results in symptom resolution in 67–89% of cases compared to 0–48% spontaneous resolution [8]. A recent prospective observational study [9] found that patients attending a physiotherapist-led vestibular rehabilitation service within emergency and acute services experienced significantly reduced dizziness and vertigo and significantly improved mobility (sustained for three months post-discharge). A validated vestibular screening tool has also been developed for physiotherapists working in emergency and acute contexts, which was found to have good levels of sensitivity and reliability [10].

Despite available training and expertise to effectively manage peripheral vestibular dysfunction, SVP is not routinely implemented within the ED context. No previous studies have evaluated the implementation process of SVP in the ED or its longer-term sustainability. Evidence around the role of ED physiotherapists in providing evidence-based BPPV management remains at an early stage of development [11] but suggests this could be an effective and acceptable approach to this issue. A greater understanding of the implementation processes and strategies required to adopt this clinical innovation is urgently needed to ensure its wider dissemination occurs effectively and efficiently.

This study aimed to complete a mixed-methods formative process evaluation of feasibility testing for SVP in an ED from the clinician’s perspective.

## Materials and methods

### Design

Ethics approval to conduct this study was sought and received from the local Human Research Ethics Committee (HREC/18/WH/120). This study utilised a hybrid trial type I design [12] with mixed-methods evaluation to build workforce capacity in the use of evidence-informed interventions and explore not only “what works” but “why” [13]. The study reported here addressed the secondary aim of understanding the context for implementation. In contrast, the primary objective of testing the feasibility of physiotherapy for BPPV in the ED was addressed by a separate pilot study [14]. An academic with implementation expertise (DH) joined the research recipients to provide the research expertise for this design, as these studies often require more resourcing than non-hybrid designs [12].

To ensure the study drew on established implementation theory, the Integrated Promoting Action on Research Implementation in Health Services (i-PARiHS) framework was used as an organising theoretical structure. The i-PARiHS framework is designed to guide the implementation of evidence-based practice. It consists of four interacting domains—the characteristics of the setting or context (Context), how evidence is facilitated (Facilitation), the individuals and recipients engaged in implementation (Recipients) and the quality and type of evidence (Innovation) [15].

### Context

SVP was implemented in the ED of a tertiary metropolitan Australian health service. This study was undertaken at a single hospital site, which provides acute and community-based services to aged, adult, paediatric and maternity populations. Approximately 250 patients present to the hospital’s ED daily, with 6.5% of those presenting with dizziness symptoms [16]. The ED treats both children and adults as a mixed urban ED, but patients with severe injuries are transferred to other hospitals housing the city’s central trauma units. Upon arrival, patients are screened using a nationally recognised triage scale and seen by the medical staff in priority order. Physiotherapy already had a presence in the ED as part of the multidisciplinary Immediate Response Service, which facilitates screening and rapid discharge for patients with complex needs.

### Clinical innovation description

**Fig. 1:**
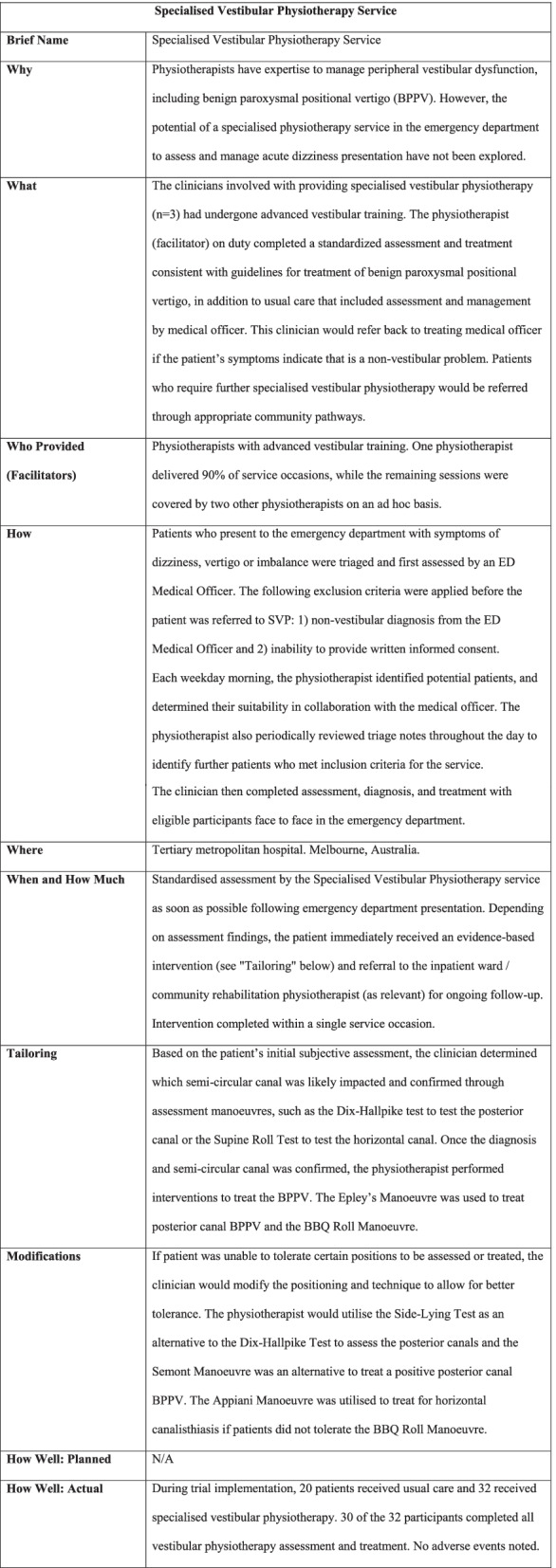
Description of the specialised vestibular physiotherapy service (based on the TiDiER Checklist) [17]

The innovation introduced in this study was having physiotherapists routinely assess and treat people with BPPV, rather than the previous model of medical intervention only. The SVP service was implemented over the second half of the 16-week recruitment period for the feasibility study. Due to available resources, it was open to eligible patients during weekday business hours (8 am to 4 pm). The SVP service was an addition to existing physiotherapy services, which provided appropriately skilled clinicians and new procedures for referral and treatment. One physiotherapist delivered 90% of the service occasions, with two other physiotherapists filling in ad hoc. All aspects of the SVP service were offered within the ED clinical space. Full details of the SVP service are presented as per the Template for Intervention Description and Replication (TIDieR) Checklist in Fig. 1.

### Implementation strategy

The implementation strategy’s goal was to make the SVP service a visible and recognisable component of the ED service and ensure its routine and sustained integration in the care pathway [12]. Targeted funding was acquired via an internal health service grant, which facilitated the creation of a new clinical service to deliver the SVP service on a trial basis [18]. The strategy utilised several interconnected strategies, beginning with extensive consultation and dissemination with the multidisciplinary ED recipients, to build an engaged coalition of partners and develop appropriate and feasible clinical innovation pathways and processes [12, 18]. The consultation phase included educational meetings, consensus discussions, multidisciplinary input into the SVP service design and the collaborative development or selection of tools for quality monitoring (such as formal referral pathways, protocols and outcome measures) [18].

As trial implementation commenced, interactive problem solving was employed to address unforeseen issues or challenges, along with regular consultation with partners and timely review of outcome measures [18]. The building and maintenance of relationships with partners was also a vital feature of the implementation strategy and was facilitated by timely feedback on the trial’s progress, prioritising contact with local opinion leaders, collection and application of patient feedback and the provision of clinical innovation specific supervision [18]. The physiotherapy department provided almost all resources required to deliver the innovation, supplemented by ad hoc consultation with medical staff regarding patient suitability and diagnosis (for which resource sharing agreements were arranged during the consultation phase) [18].

Two members of the study recipients (KI, DH) had no direct role in SVP service delivery or implementation. At the same time, the other two (ML, AL) delivered the assessments and innovations and were directly involved in the trial. The SVP clinicians were also members of the study recipients because the potential success of SVP service implementation depended on their ability to integrate it with the existing ED services they were also providing. Embedding the clinicians within the study recipients enabled effective care coordination but was also recognised as a potential source of bias within the process evaluation. The full range of outcomes from the SVP service evaluated in the feasibility study [14] is provided in Additional file 1.

### Eligibility and recruitment for implementation study

Purposive sampling was undertaken to gather data from clinicians with direct experience in implementing the SVP service in the ED at the study site. Eligible participants included physiotherapists providing the SVP service, nursing and medical clinicians from the ED directly involved with triaging and assessment, and inpatient acute care and community rehabilitation clinicians impacted by the trial implementation of the SVP service due to inpatient admission or community referrals. Due to their crucial role in its design and implementation, study team members who met these criteria (ML, AL) were invited to participate. Participants included both senior and junior staff to capture the diverse perspectives, and the multidisciplinary scope of the study was emphasised throughout the recruitment phase.

Participants were able to indicate on the consent form what modes of data collection they wished to participate in (i.e. measure only, interview/focus group only, both). Several options for participation were offered to allow as many staff to participate as possible in the context of variable availability and competing clinical demands. The sample size was informed by purposive selection, identifying participants with direct experience of SVP implementation. The sample population included 13 staff members (4 care coordinators, three doctors, five physiotherapists, and one nurse), and theoretical saturation was not an appropriate goal in this small, single-site study.

### Characteristics of implementation

Organisational readiness for change and clinician perceptions of the implementation process (including acceptability, appropriateness, feasibility and context) were the key characteristics of interest. Four measures were used—the Organisational Readiness for Change Assessment (ORCA) [19], Acceptability of Intervention Measure (AIM), Intervention Appropriateness Measure (IAM) and Feasibility of Intervention Measure (FIM) [20].

The ORCA is a 77-item checklist designed to operationalise the constructs of the i-PARiHS framework [19]. The psychometric properties of the ORCA have been established, with acceptable reliability and validity found across most sub-scales (except those related to evidence). The ORCA was used retrospectively to enable clinicians to identify essential factors in SVP implementation.

The AIM/IAM/FIM is a combined suite of implementation measures that monitor and evaluate the implementation success. These scales have achieved reasonable structural validity, known group validity, test–retest reliability and sensitivity to change [20]. Higher scores indicate greater acceptability, appropriateness and feasibility, and scale scores are calculated with mean responses.

Qualitative data was collected via semi-structured interviews and focus groups. A bespoke set of prompts were developed (see Additional file 2), based upon previously published reflective questions for facilitators using the i-PARiHS framework [15]. These interviews and focus groups were facilitated by a study team member with no direct role in SVP service delivery or implementation (DH). The interviews and focus groups began with specific prompts about assessment and treatment components in the clinical practice guidelines before encouraging a more general reflection on the implementation process. All focus groups and interviews were digitally recorded and transcribed verbatim by an external contractor for analysis.

### Data analysis

A convergent parallel model of mixed methods was applied, which utilises concurrent collection of quantitative and qualitative data and equal valuation when formulating an overall interpretation [21].

The ORCA is already aligned to i-PARiHS domains; however, items on the AIM/IAM/FIM were also categorised into the i-PARiHS to enable a holistic and theoretically informed approach to integrated analysis. This alignment was undertaken by reviewing the definitions and descriptions of the four domains (Context, Facilitation, Recipients and Innovation) and assigning all items on the AIM/IAM/FIM to the Recipient domain (see Additional file 3). This alignment is related to the conceptualisation of perceived acceptability, appropriateness and feasibility as determinants of individual engagement. One researcher (DH) undertook alignment before being independently reviewed and confirmed (KI). This data was analysed using SPSS version 25.0, and data from all measures were maintained in their item form. Responses to each item are reported using descriptive statistics, namely proportions.

Qualitative transcriptions were subjected to a priori thematic analysis [22], also aligned to the i-PARiHS framework. A codebook was developed to ensure analytical consistency, including definitions and examples for all i-PARiHS framework concepts (see Additional file 4). Transcriptions were systematically coded and categorised to a theme within each domain. Two researchers (KI, DH) independently reviewed each transcript and assigned codes to sections of the text based on the definitions provided in the codebook. A third researcher (ML or AL) also reviewed all coding, with a few instances of disagreement resolved by consensus. Within the a priori themes provided by the i-PARiHS framework, the codes were then compared to identify key themes within each domain and any links between codes classified under different domains.

Both quantitative and qualitative findings for each i-PARiHS domain were then integrated for the final mixed-methods analysis. Within each domain, the study recipients sought to identify the areas of agreement and disagreement between the quantitative and qualitative findings to provide integrated results based on both forms of data [23].

## Results

Nine participants (69% of sample population) responded to the measures, six participated in a focus group and one chose to be interviewed due to conflicting shift schedules.

### Facilitation

**Fig. 2:**
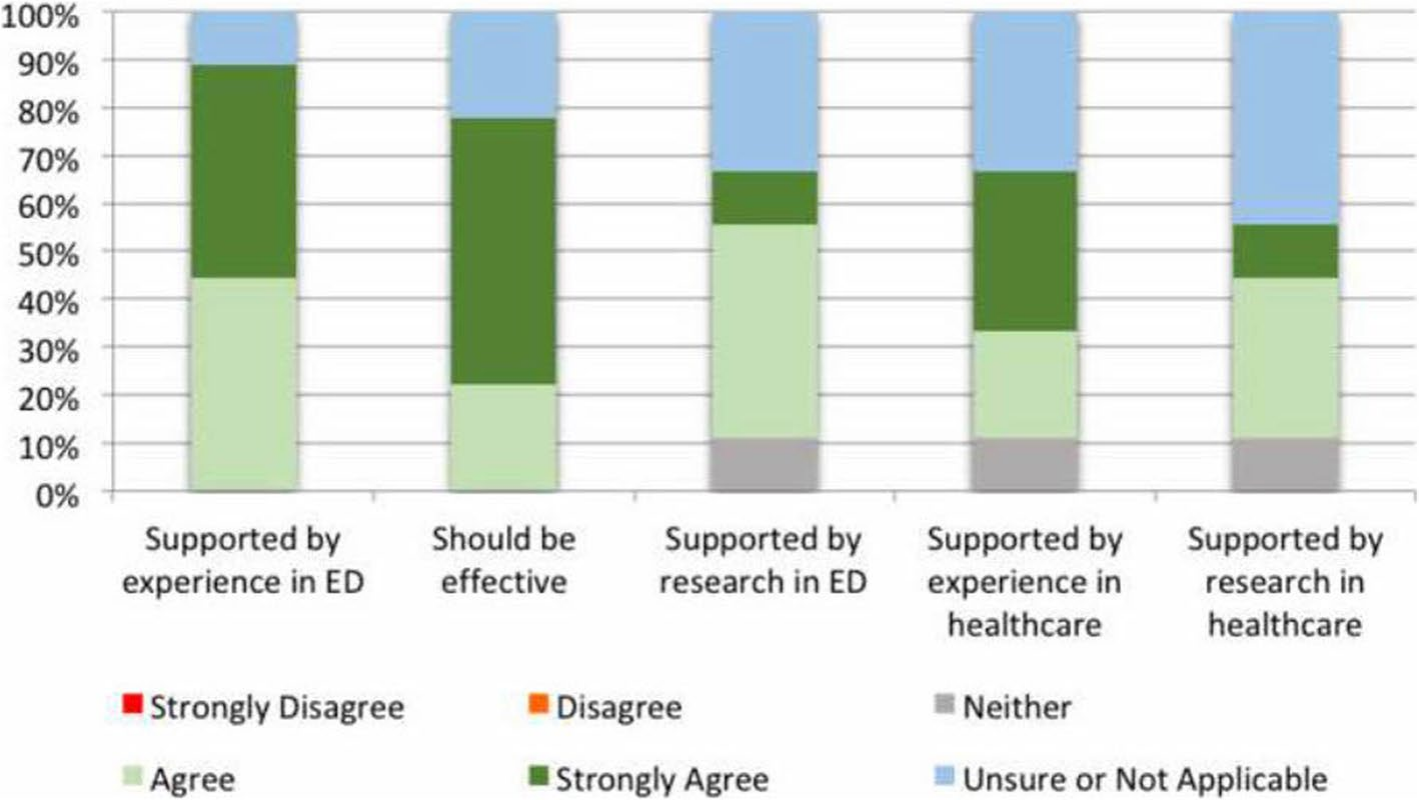
Participant perceptions of how evidence was facilitated for the specialised vestibular physiotherapy

As shown in Fig. 2, clinicians agreed or strongly agreed in their ORCA responses that senior leadership practices and champions positively impacted the implementation of SVP in the ED. The SVP service clinicians were senior and experienced physiotherapists who had the clinical competency and confidence to educate and advocate for SVP to ED staff. The multidisciplinary recipients involved in the trial funding and ethics applications were also enthusiastic about the service and developed a sense of ownership to act as “champions” for SVP [24]. However, there was more uncertainty around general leadership roles for the overall trial, implementation progress, implementation communication and evaluation plans.

Participants especially highlighted a need for exceptional communication and interpersonal skills; “it really came down to [facilitator – SVP clinician] ability to communicate and develop rapport with the multidisciplinary team down in ED”. The ability to clearly and effectively communicate across multiple audiences and purposes was also required, as this skill was essential to all phases of implementation (i.e. planning, trial commencement, ad hoc problem solving, reflection and evaluation).

### Innovation

**Fig. 3:**
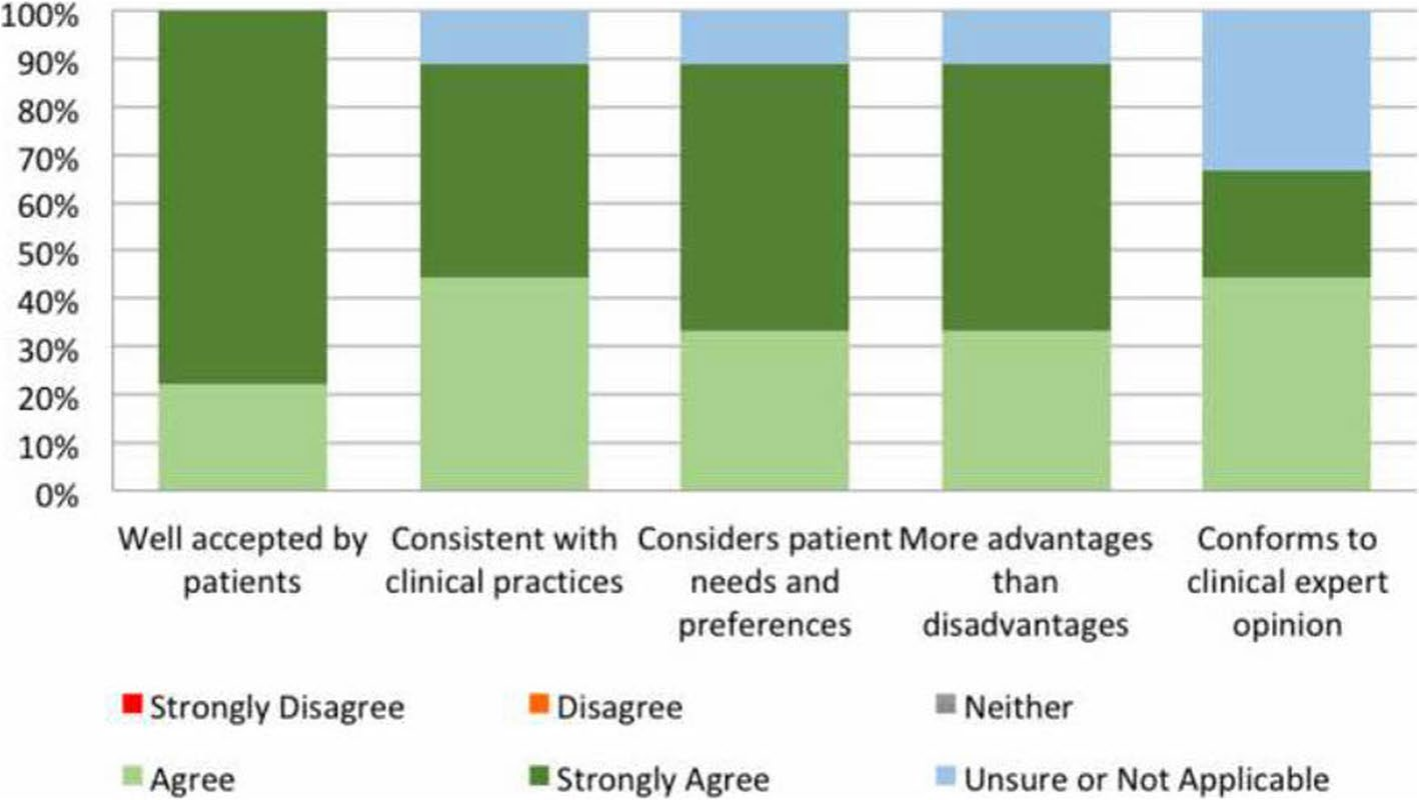
Participant perceptions of specialised vestibular physiotherapy

The majority of ORCA responses (*n* = 6, 66.7%) indicated the evidence for SVP for BPPV was perceived to be very strong, and SVP was perceived as fitting the priorities of the service and needs very or extremely well (*n* = 6, 82.7%). As shown in Fig. 3, all participants believed the SVP service successfully addresses and treats BPPV.

The SVP service was generally perceived as positively impacting access and flow in the ED; “there was someone there who could actually do the treatment, and that meant free them up maybe to do something else”. Participants experienced significant levels of satisfaction with the implementation process, which was not perceived as burdensome or overly difficult and enabled a proactive (rather than reactive) approach; “I really enjoyed being able to treat patients using evidence-based practice, doing things that we know work and actually make a difference”.

The interventions used by SVP have multiple and potentially confusing names. They often need additional explanations to colleagues and patients because “it looks a bit awkward. It looks strange, and there’s a lot of education that goes around that”. Working with culturally diverse patients also presented consistent challenges; “the terms delirium, dizziness, light-headedness or vertigo can be used interchangeable, depending on your cultural background, your belief systems, your health literacy”. The sporadic nature of BPPV presentations to the ED was identified as a potential limitation but was addressed in this trial by having physiotherapists float for other services when not required in ED; “We had weeks where we’d have eight or nine patients, and then weeks where we’d have none, so juggling not being able to predict was an added stress”.

### Recipients

**Fig. 4:**
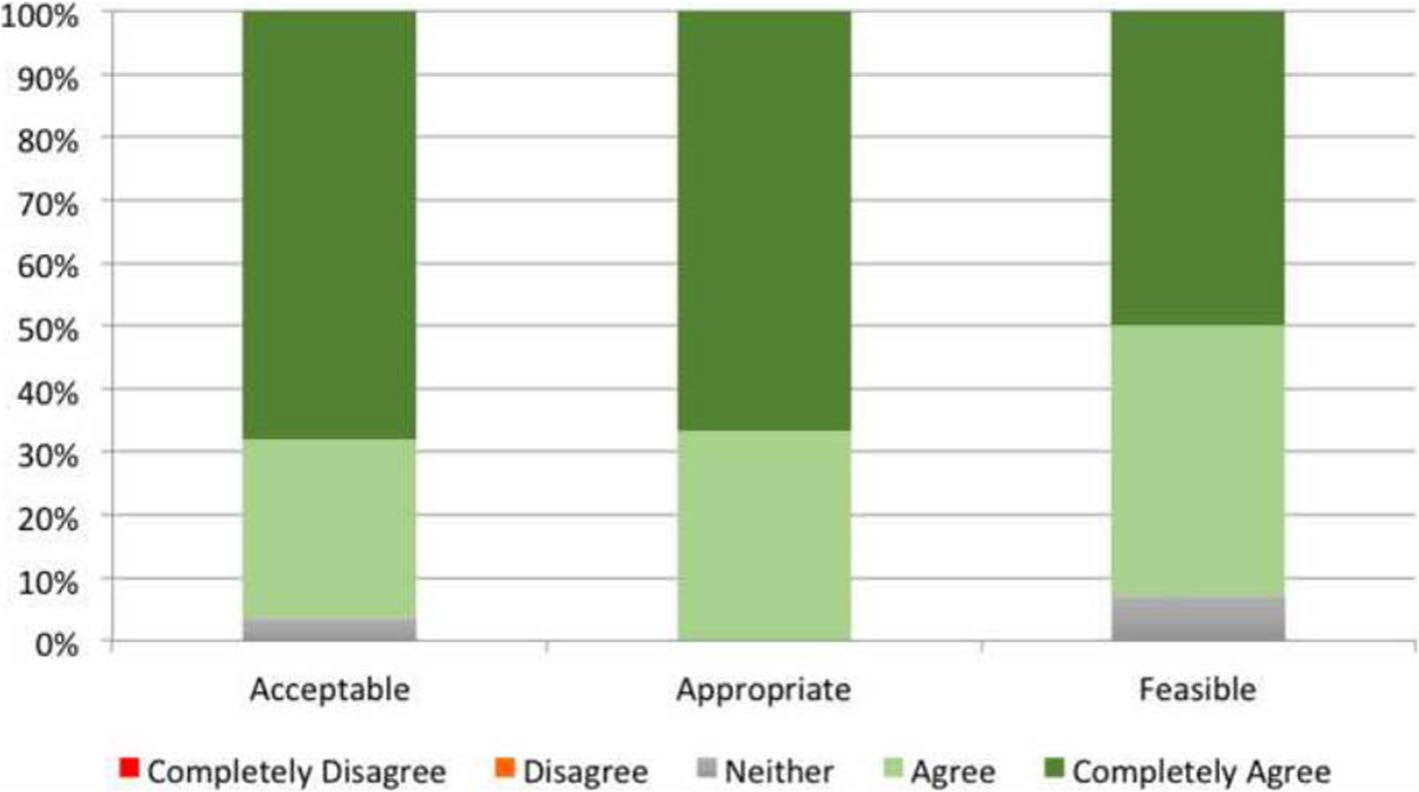
Recipient perceptions of the acceptable, appropriateness and feasibility of specialised vestibular physiotherapy

Most AIM/IAM/FIM responses stated that the SVP service was appropriate, acceptable and feasible in the ED (see Fig. 4).

The successful implementation of SVP relied upon the formation of good relationships with colleagues, especially given the novelty of the SVP service; “Having to re-establish identity as who you are and what your skills are and to work out who people are to be able to have that relationship to go from”. These working relationships assisted with obtaining the understanding and buy-in of the service from senior ED staff, which translated to more junior staff’s acceptance of its implementation. The ED medical recipients were acknowledged as crucial stakeholders in the implementation success, with their enthusiasm and investment an important facilitator; “The ED doctors seem to be buying in … we certainly needed buy-in from the key physicians, so neurology, general medicine, had a multidisciplinary input”.

However, a general lack of knowledge and skill within physiotherapy was consistently perceived as a barrier to scaling up SVP. While formalised continuing professional development is available, experiential learning remains a common path to competence; “I’ve never done a specialised course, I really learned from seeing clinicians, or from doing doubles and stuff”. Variable practice was also observed amongst other colleagues, suggesting a more widespread need for capacity building; “I have seen the most weird, I wouldn’t say wonderful, variations of Hallpikes … your interpretation of results and what you do with it is not all on the same page”. The few physiotherapists delivering the SVP service continued the concentration of this knowledge in a handful of clinicians, but a plan to disseminate these professional skills more widely is being developed.

### Context (local, organisational and external health system)

**Fig. 5:**
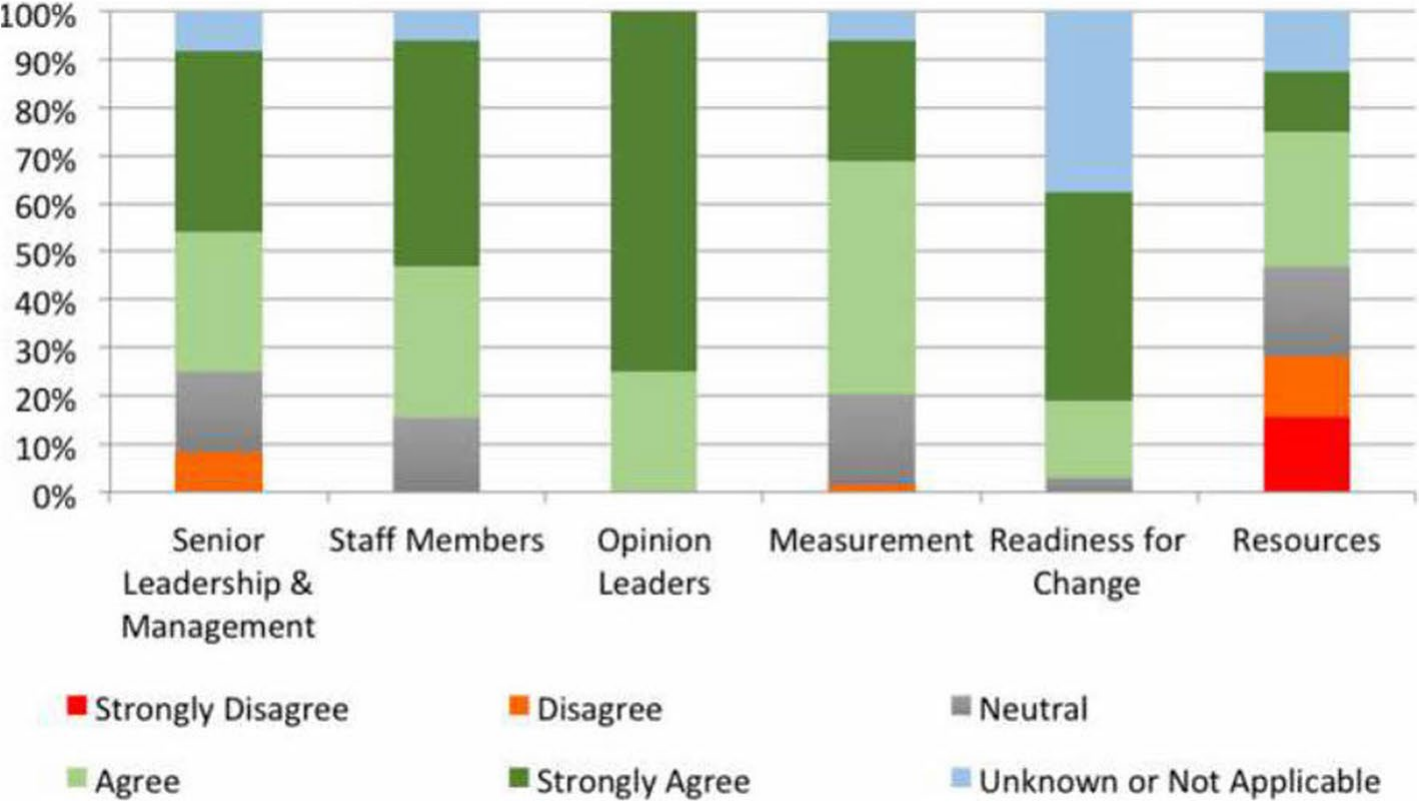
Participant perceptions of the context for specialised vestibular physiotherapy

According to ORCA responses, the local ED recipients and broader organisations were perceived as committed and receptive to enabling access to this effective treatment (see Fig. 5). Clinical leaders in the department were essential to the implementation process as they helped establish team goals and provided timely feedback. However, more ambivalent views were expressed about the organisation’s ability to provide adequate resources to sustain the SVP service.

Practical measures required to facilitate an ongoing SVP service were identified, including developing greater clarity around referrals and pathways; “For a goal-based service, you need to have this information if you’re going to refer to us”. Inclusion of the service in organisation-wide policies or regulations and the provision of targeted education around SVP was also considered an essential resource for sustained implementation: “I think as an organisation we need to look more broadly at how it would fit in, so it’s not just this little service that operates in ED”.

To provide the SVP service within existing finite resources, some participants suggested a reconfiguration of the broader physiotherapy department would be required; “withdraw our services from elsewhere when they’re not necessarily being used that effectively, or what extra resources we would need to be able to run that kind of program”. This could enable the service to be offered seven days a week, ensuring all patients presenting with BPPV had equal access; “It needs to be a seven-day service … because obviously dizziness happens seven days a week”.

The role of general practitioners following contact with the SVP service also emerged as a strong theme in the data. While some were supportive of their patients receiving specialised physiotherapy, others did not perceive it as an appropriate innovation; “People were seen then went back to the GP and the GP said ‘that’s a load of rubbish. It’s complete rubbish, don’t bother doing that. It’s wrong’”. As a result, forming partnerships with community providers was highlighted as an essential future priority.

## Discussion

The findings of this study have provided a clinician’s perspective on implementing SVP in an emergency department. The i-PARiHS framework describes successful implementation as a function of facilitation (innovation + recipients + context) [25]. Successful implementation is demonstrated by (1) achievement of the implementation goal; (2) the innovation being embedded in practice; (3) engaged and motivated individuals, teams and stakeholders; and (4) minimal variation across contexts. While the fourth benchmark did not apply to this study, the findings presented here support the other identified markers of success.

A variety of barriers and facilitators to the implementation process were identified by participants, some of which spanned multiple domains of the i-PARiHS framework. For example, the crucial role of service leaders and champions with excellent interpersonal skills in enabling the implementation of the SVP service was discussed in relation to both facilitation and context. Service leaders significantly impact their colleagues’ attitudes, priorities and behaviours relating to SVP [26], and this finding consolidates existing evidence in this area. The use of champions has also been found to enable successful implementation in healthcare [27]; however, similar to previous innovations, the position was not operationalised formally in this study.

The SVP service would need to be embedded into a range of organisation-wide policies and procedures, including (but not limited to) practices relating to access, referral pathways, triage processes, treatment spaces and occupational health and safety. Developing recruitment and physiotherapy workforce resources to facilitate this service will also be critical to its ongoing sustainability. It could be achieved via a business case for new positions and/or redistribution of existing resources.

The medical staff were particularly identified as key contacts within and beyond the ED department, reflecting their core role within the Australian health system. Their influence on the implementation process could be positive or negative, depending on their perception of the value and evidence base for SPV. The impact of workplace cultures on hierarchical relationships in acute health is well recognised. Still, it may be a barrier to implementation when they result in the formation of silos or dysfunctional care pathways [28]. The medical staff (and nursing staff) were gatekeepers for this service, as they triaged and determined whether appropriate patients were referred for assessment and treatment. This is a common feature of the allied health practice context and demands multidisciplinary collaboration, which adds to the complexity of every innovation [29]. The findings of this study indicated that these relationships were successfully negotiated during this implementation process.

The ED recipients and other stakeholders were also reported to accept and support the new service, which was attributed to a shared recognition of the potential for positive outcomes for patients and the ED itself. The quantitative and qualitative data were also consistent in supporting the perceived acceptability, appropriateness and feasibility of the SVP service in the ED. Developing a shared vision or shared goals for change is an effective strategy for promoting buy-in and participation whenever practice transformation is attempted [30]. However, in this study, many participants reported feeling unsure about the implementation and evaluation plans for SVP in the ED, despite these being clearly articulated in the funding and ethics applications associated with the trial. Therefore, investment from the multidisciplinary recipients might be consolidated or developed even further through more detailed reporting of goals, planned implementation strategies and evaluation results in the future.

A key barrier identified in the study was varying levels of knowledge of local ED staff around the role of physiotherapy with these patients and the effectiveness of the innovations themselves. This lack of awareness and differing opinions on the scope of practice may lead to missed opportunities for referrals and less than optimal execution of the SVP service. Some physiotherapists also described a lack of confidence around providing these assessments and innovations, as they have not received formalised training. A lack of capacity within the physiotherapy profession may limit the potential scaling up of these services, as there will not be sufficient staff available to deliver the service. A previous evidence synthesis has found that educational strategies may effectively promote implementation, but only if they directly address barriers and facilitators relevant to the innovation [31]. Therefore, the findings of this study could provide a basis for the design of two tailored education packages: one to build capacity in assessments and treatments for physiotherapists and another to focus on the implementation of these services for all partners.

In summary, the findings of this study indicate several key recommendations for the future implementation of SVP in the ED. Investment in the formation and maintenance of relationships with all stakeholders will enhance the chances of success, particularly senior leadership, champions and medical colleagues. A shared vision of the goals and outcomes of these services for both patients and the ED service itself should be explicitly stated and form the basis of ongoing evaluation to ensure this innovation meets the needs of its stakeholders. Ensuring that goals and outcomes are explicit should also focus on consistent terminology (for use with both patients and colleagues) and the availability of or need for resources. Efforts to increase capacity within services for SVP should be tailored to meet the different needs of the physiotherapy workforce and multidisciplinary recipients. Finally, formal processes, procedures and policies to embed the service into existing organisational systems (such as position descriptions and referral pathways) should be developed as early in the implementation process as possible and regularly reviewed by all stakeholders.

### Strengths and limitations

This study has several strengths, beginning with collecting data from participants across different disciplines within the ED. This provided a holistic perspective of the success of the implementation of the service and facilitated an understanding of the process from multiple points of view. A rigorous approach to the qualitative analysis was also undertaken, including multiple coding and peer review. Using the i-PARiHS framework in both methodological design and analysis also ensured the study retained its focus on implementation and explored the process comprehensively and accurately.

However, the small sample size in this study also introduces some significant limitations to the interpretation of the findings. Most participants were physiotherapists during the focus groups and interviews, despite an open invitation to other stakeholders. Therefore, this data may not represent all stakeholders’ perceptions and did not reach theoretical saturation.

The lack of nursing participants may reflect their varied shift patterns, contributing to less consistent exposure to the SVP service. This study is also only reflective of the implementation process at a single campus of a single health service within the Australian health service context. Finally, retrospective data collection may have missed changes in perception and experience over time during the implementation process.

### Implications

This study provides preliminary data on the implementation process for SVP in ED contexts and demonstrates that this is a complex and sometimes challenging experience for recipients. As the first study to address this aspect of the topic, it extends the existing evidence base in a new direction that is important to the practical development of these services. This study also provides a basis for future research, education and practice as a guide for clinicians and service providers as this innovation is scaled up. The recommendations presented above can assist physiotherapists and their colleagues in implementing SVP more effectively after adapting and modifying the innovation to meet their local needs.

## Conclusion

The findings of this study show that the process of implementation of an SVP team in an ED context was generally perceived positively by recipients. Recommendations for future implementation and development were also identified, including building relationships with stakeholders through education sessions; developing an explicit shared vision; explicitly stating goals and intended outcomes; embedding the innovation in organisational processes, procedures and policies with clear referral pathways and SVP scope of practice; and increasing workforce capacity to delivery to support SVP for patients with BPPV presenting to the ED. The outcomes of this study indicate that this innovation has significant potential to make a meaningful impact on both the function of the ED and the lives of patients presenting with dizziness.

## Supplementary Information

**Additional file 1.** Outcome of Specialised Vestibular Physiotherapy.

**Additional file 2.** ﻿Open Questions used in semi-structured interviews and focus groups.

**Additional file 3.** Alignment of ORCA and AIM/IAM/FIM quantitative items with domains of the PARHiS Framework.

**Additional file 4.** Implementation in the ParHIS Framework.

## Data Availability

The datasets used and analysed during the current study are available from the corresponding author on reasonable request.

## Abbreviations

AIM: Acceptability of Intervention Measure
BPPV: Benign paroxysmal positional vertigo
CRT: Canalith repositioning technique
DHT: Dix-Hallpike test
ED: Emergency department
FIM: Feasibility of Intervention Measure
IAM: Intervention Appropriateness Measure
ORCA: Organisational Readiness for Change Assessment
i-PARiHS: Integrated Promoting Action on Research Implementation in Health Services framework
SRT: Supine roll test
SVP: Specialised vestibular physiotherapy
