# Supplementary material for: Implementing specialised vestibular physiotherapy in an emergency department: a process evaluation
Source: Implement Sci Commun. 2022 Jun 11;3:63. doi: 10.1186/s43058-022-00313-2 (PMC9188154; doi:10.1186/s43058-022-00313-2)
Supplement: Supplementary file 1 — Additional file 1. Outcome of Specialised Vestibular Physiotherapy. [file 43058_2022_313_MOESM1_ESM.docx]

**Additional File 1: Outcome of Specialised Vestibular Physiotherapy**

Feasibility

Primary Outcome:

- Total number of patients presenting to the ED with one of the target symptoms documented at triage that are appropriate, willing and able to participate in vestibular physiotherapy

Secondary Outcomes:

- Total number of patients who successfully completed each component of the assessment.
- Reasons for non-completion of any assessment component.
- Direct costs associated with provision of the Dizzy-PT service measured by clinician time.
- Total number of patients requiring an interpreting service.

Effectiveness

- Total number of patients presenting with target symptoms who received a “gold-standard” DHT and/or SRT in the ED.
- Total number of patients with a documented positive test result for BPPV.
- Total number of patients with a positive test result for BPPV treated with a “gold-standard” CRT.
- Total number of patients undergoing CTB or MRI-B
- ED length of stay (hours)
- Proportion of patients requiring admission to an inpatient ward, and length of that admission
- Proportion of patients requiring specialized medical physician outpatient follow-up (ie. other than GP)
- Patient satisfaction with clinical care provided measured on a 10-point Visual Analogue scale in response to each of the following questions:
- Overall, how satisfied were you with your care in the ED?
- How satisfied were you with care provided by your physiotherapist (if relevant)?
- How satisfied were you with the information provided to you about your condition and its ongoing care?
- Number of patients returned to usual activity levels at 5-days post-presentation to ED.
- DHI-score at 5-days post-presentation to ED.
- Representations to ED within 5 days
