# Supplementary material for: Implementing specialised vestibular physiotherapy in an emergency department: a process evaluation
Source: Implement Sci Commun. 2022 Jun 11;3:63. doi: 10.1186/s43058-022-00313-2 (PMC9188154; doi:10.1186/s43058-022-00313-2)
Supplement: Supplementary file 2 — Additional file 2. ﻿Open Questions used in semi-structured interviews and focus groups. [file 43058_2022_313_MOESM2_ESM.docx]

Open Questions

1. Please describe your understanding of the evidence base behind specialised vestibular physiotherapy (SVP)? Prompts – Strengths / weaknesses, consensus across disciplines

1. Please describe your personal knowledge and skills related to SVP? Prompts – Strengths / weaknesses, how were they developed?

1. We will begin by going through each specific components of the guideline mediated assessments and treatments. For each of these components, did you experience any particular barriers or challenges (i.e. environmental, patient related [symptoms, health literacy, refusal etc], other clinicians, logistics [time, service catchments, waiting lists])

- Hallpike
- Supine Roll Test
- CRTs
- Obtaining thorough subjective assessment
- Occulomotor tests (including HIT, active VOR, skew eye)
- Provision of patient education
- Organising follow-up assessment
- Prescribing / administering symptom control medications
- Identifying relevant medical history
- Mobility and function assessment
- Teaching of habituation/adaptation exercises

The following questions will take a more general perspective of the implementation of SVP, to gain an overview of your experiences.

1. Who were the key stakeholders in the implementation of SVP?

1. How did you experience your role in the trial while it was happening? Prompts – Feedback, formative data

1. What changes to your prior practices had to be made to implement SVP at Western Health?

1. What are the advantages of SVP? What facilitates its delivery?

1. What are the disadvantages of SVP? What barriers are there to its delivery?

1. What sort of leadership and other support did you experience around the implementation of SVP?

1. How sustainable will SVP be into the future? What would you like to see change about how it is implemented?
