# Supplementary material for: Implementing specialised vestibular physiotherapy in an emergency department: a process evaluation
Source: Implement Sci Commun. 2022 Jun 11;3:63. doi: 10.1186/s43058-022-00313-2 (PMC9188154; doi:10.1186/s43058-022-00313-2)
Supplement: Supplementary file 3 — Additional file 3. Alignment of ORCA and AIM/IAM/FIM quantitative items with domains of the PARHiS Framework. [file 43058_2022_313_MOESM3_ESM.docx]

Additional file 3: Alignment of ORCA and AIM/IAM/FIM quantitative items with domains of the PARHiS Framework.

| Domain & Section | Items (Abbreviated) | Measure |
| --- | --- | --- |
| Facilitation | |  |
| Senior Leadership Practices | - Proposal of SVP appropriate and feasible - Clear goals for improving patient care - Appropriate schedules and deliverables established - Designated clinical champion identified | ORCA |
| Champion Characteristics | - Accepted responsibility for success - Authority to carry out implementation - Considered a clinical opinion leader - Worked well with the intervention team and providers | ORCA |
| Leadership Implementation Roles | - Agreed on the goals - Were informed and involved - Agreed on adequate resources - Set a high priority on success | ORCA |
| Implementation Team Members | - Share responsibility for success - Clearly defined roles and responsibilities - Released time or able to accomplish implementation within usual caseload - Had support and other resources required | ORCA |
| Implementation Plan | - Identified specific roles and responsibilities - Clearly described tasks and timelines - Appropriate provider / patient education - Acknowledge staff input and opinions | ORCA |
| Implementation Communication | - Regular project meetings - Involvement of quality management staff - Regular feedback to clinical management - Regular feedback to clinicians | ORCA |
| Measuring Implementation Progress | - Feedback from patients - Feedback from staff - Developing and distributing regular performance measures - Forums for presentation / discussion | ORCA |
| Implementation Resources | - Staff incentives - Equipment and materials - Patient awareness / need - Staff buy-in - Intervention team - Evaluation process / procedure | ORCA |
| Evaluation and Improvement Plans | - Periodic outcome measurement - Staff participation / satisfaction survey - Patient satisfaction survey - Dissemination plan for performance measures - Review of results by clinical leadership | ORCA |
| Innovation | |  |
| Strength of Evidence | - Participants opinion - Clinical experts opinion | ORCA |
| SVP | - Supported by research evidence in the ED - Supported by research evidence in healthcare - Effective based on current knowledge - Supported by clinical experience in the ED - Supported by clinical experience in healthcare - Conforms to the opinions of clinical experts - Well accepted by patients - Consistent with practices accepted by patients - Considers needs and preferences of patients - More advantages than disadvantages - Overall, how well does SVP address or treat BPPV? | ORCA |
| Recipients | |  |
|  | - Acceptability - Appropriateness - Feasibility | AIM  IAM  FIM |
| Context (Local & Organisational) | |  |
| Context (Local) | - Staff members - Clinical leaders - Opinion leaders | ORCA |
| Context (Organisational) | - Rewards clinical innovation and creativity - Solicits opinions of clinical staff - Seeks ways to improve patient education and increase patient participation - General resources | ORCA |

Note: SVP = specialized vestibular physiotherapy.
