# Supplementary material for: Implementing specialised vestibular physiotherapy in an emergency department: a process evaluation
Source: Implement Sci Commun. 2022 Jun 11;3:63. doi: 10.1186/s43058-022-00313-2 (PMC9188154; doi:10.1186/s43058-022-00313-2)
Supplement: Supplementary file 4 — Additional file 4. Implementation in the ParHIS Framework. [file 43058_2022_313_MOESM4_ESM.docx]

Successful Implementation in the ParHIS Framework is defined as:

**SI = Fac^n^(I + R + C)**

SI = successful implementation

- Achievement of agreed implementation/project goals
- The uptake and embedding of the innovation in practice
- Individuals, teams and stakeholders are engaged, motivated and ‘own’ the innovation
- Variation related to context is minimised across implementation settings

Fac^n^ = facilitation

I = innovation

R = recipients (individual and collective)

C = context (inner and outer)

| **Innovation** | **Harvey & Kitson unless indicated otherwise.** |  |
| --- | --- | --- |
| **Key Characteristics** | **Definition** | **Example** |
| Underlying knowledge sources | How the evidence for physio for BVP was derived, its rigour and robustness. | The studies and guidelines that support physio for BVP |
| Clarity | The clarity of the innovation being implemented – is it clear what to do and when? (No formal definition available) |  |
| Degree of fit with existing practice and values (compatibility or contestability) | Does physio for BVP fit in the local setting, how much change is needed in processes and thinking, and how likely is it to be accepted or contested by the people who need to apply it? | Examples of changes to existing practices or documentation to accommodate physio for BVP |
| Usability | The accessibility and useability of the evidence upon why physio for BVP (i.e guideline, care pathway). How easy is it to understand what has to be done? |  |
| Relative Advantage | The perceived advantages physio for BVP offers over the current ways of doing things. | Comparisons to previous ways of treating these patients |
| Trialability | The potential of physio for BVP to be tested out / piloted on a small scale in the first instance | References to the scope of this trial and how it could be extended |
| Observable Results | The outcomes seen from the implementation of physio for BVP | Perceptions of staff about the outcomes |

| **Recipients (Staff)** |  |  |
| --- | --- | --- |
| **Key Characteristics** | **Definition** | **Example** |
| Motivation | How much individuals, teams and organisations want to apply the change in practice | Need to encourage people, or ‘investment / buy in’ from stakeholders |
| Values and beliefs | The perceptions of physio for BVP as valuable and worthwhile, or useless / not required. |  |
| Goals | How physio for BVP aligns with individual, team and organisational goals. Also, the goals of the implementation process. | Why was physio for BVP being done? |
| Skills and knowledge | The existing or developed capacity of recipients to understand and deliver physio for BVP | Training regime for the physios |
| Time, resources, support | The resources (both tangible and intangible) required to implement physio for BVP | All other resources needed to deliver physio for BVP |
| Local opinion leaders | Identifying the local opinion leaders, and their approach to physio for BVP (i.e. supportive or obstructive) | Who were the key people in ED and beyond? What was their attitude? |
| Collaboration and team work | Instances of inter-professional collaboration and team work relevant to physio for BVP | Who works with the physios? |
| Existing networks | Use of pre-existing social networks within and beyond WH for physio for BVP |  |
| Power and authority | Power hierarchies, power and permission required to implement physio for BVP | The role of the doctors as gatekeepers |
| Presence of Boundaries | Boundaries (between teams, services etc) which are relevant to physio for BVP | Transitions in care – i.e. entry to ED or referral on |

| **Context (Local)** |  |  |
| --- | --- | --- |
| **Key Characteristics** | **Definition** | **Example** |
| Formal and informal leadership | Individuals in designated positions “…at any level of the organization including executive leaders, middle management, frontline supervisors, and team leaders, who have a direct or indirect influence on the implementation." | Who were in positions of leadership? May or may not be facilitators. |
| Culture | “the way things are done around here”; or “prevailing values and beliefs as a prerequisite to introducing and sustaining change”[10] Also the way people believe things are or can be done around here, whether it is accurate or not. | ED culture – how are / were things done |
| Past experiences of innovation and change | Experience within WH of introducing changes at the local level in the past, which could have an influence on physio for BVP | Any previous experiences of change in the ED or physio |
| Mechanisms for embedding change | Mechanisms that support learning, evaluation and practice change such as team meetings, audit and feedback processes, PD opportunities and performance reviews | Practices / structures which get it into practice and keep it there |
| Evaluation and feedback processes | Evaluation of the physio for BVP project, and also opportunities within it to receive feedback while it was being implemented | Any previous experiences of change in the ED or physio |
| Learning environment | Aspects of WH (at the local level) which promote the development of capacity around practice change, such as PD, feedback, dissemination , networking and communication |  |

| **Context (Organisational)** |  |  |
| --- | --- | --- |
| **Key Characteristics** | **Definition** | **Example** |
| Organisational priorities | Alignment of physio for BVP with WH strategic priorities | Best care / personal care etc |
| Senior leadership and management support | How senior leadership and management have been engaged in physio for BVP | Who was involved at the senior levels in supporting this project? |
| Culture | How the overall culture of WH supports (or not) physio for BVP | WH culture – how are / were things done |
| Structure and systems | The influence of structural systems on physio for BVP | i.e. communication / documentation, physical location, infrastructure |
| History of innovation and change | Previous experiences of change at WH that are considered relevant to physio for BVP | Any previous experiences of change at WH |
| Absorptive capacity | The capacity to recognize the value of new external information, assimilate it and apply it to commercial (therapeutic) ends' (Cohen & Levinthal, 1990, p. 128) | WHs ability to recognise new evidence and than apply it |
| Learning networks | Aspects of WH (at the organisational level) which promote the development of capacity around practice change, such as PD, feedback, dissemination , networking and communication |  |

| **Context (External Health System)** |  |  |
| --- | --- | --- |
| **Key Characteristics** | **Definition** | **Example** |
| Policy drivers and priorities | The impact of current health policy and state / national priorities for action and improvement on physio for BVP |  |
| Incentives and mandates | Incentives in the wider health system that support physio for BVP | i.e. pay for performance, hour limits for ED |
| Regulatory frameworks | Regulatory frameworks in the wider health system that impact on physio for BVP | i.e. physio scope of practice, ED regulations |
| Environmental (in)stability | Any stable or unstable aspects of the wider health system that impact physio for BVP | Changes in ED, long time staff |
| Inter-organisational networks and relationships | Collaborative networks or links between WH and other organisations that are relevant to physio for BVP | Benchmarking or collaboration with other services |

| **Facilitation** |  |  |
| --- | --- | --- |
| **Key Characteristics** | **Definition** | **Example** |
| Purpose, external and/or internal role | The purpose or aim of facilitation roles, and whether the person is internal or external to the implementation team |  |
| Expectations and activities | What others around them expect of facilitators  The skills performed by facilitators in relation to each domain: (Harvey & Kitson book)   - Characteristics of Innovation – problem identification, acquiring / appraising evidence, baseline context & boundary assessment, stakeholder mapping. - Recipients – Goal setting, consensus building, audit & feedback, improvement methods, project management, change management, team building, conflict management & resolution, barriers / boundary assessment, boundary spanning. - Inner context: Local level – local context assessment, communication & feedback, networking, boundary assessment & spanning, negotiating & procedures, structuring learning - Inner context: Organisational level – stakeholder engagement, communication and feedback, marketing and presentation, networking boundary spanning, negotiating & influencing, policies and procedures - Outer context: political awareness & influence, communication, marketing, networking, boundary spanning, sustainability & spread | What did people think the facilitators should do?  What did the facilitators actually do? |
| Skills and attributes of facilitator | The personal attributes of facilitators (i.e. facilitation style, approach, characteristics)  The personal skills of facilitators which enables them to undertake the identified activities |  |
